# The effects of blood pressure on post stroke cognitive impairment: BP and PSCI

**DOI:** 10.1111/jch.14373

**Published:** 2021-11-20

**Authors:** Yue Wang, Shiping Li, Yuesong Pan, Mengxing Wang, Xiaoling Liao, Jiong Shi, Yongjun Wang

**Affiliations:** ^1^ National Clinical Research Center for Neurological Diseases Beijing Tiantan Hospital Capital Medical University Beijing People's Republic of China; ^2^ Department of Neurology Beijing Tiantan Hospital Capital Medical University Beijing People's Republic of China

**Keywords:** hypertension, post stroke cognitive impairment, stroke

## Abstract

Cognitive function following acute ischemic stroke (AIS) is critical to prognosis and quality of life. Hypertension is a risk factor for stroke and is associated with post stroke cognitive impairment (PSCI). However, the optimal blood pressure parameters after AIS is unknown. This is a sub‐study of the Impairment of CognitiON and Sleep after acute ischemic stroke or transient ischemic attack in Chinese patients (ICONS) study conducted between August 2015 and March 2018. Cognition was assessed at two‐week (2w), three‐month (3 m), and twelve‐month (12 m) by Montreal Cognitive Assessment (MoCA). A total of 682 participants who met the inclusion criteria were enrolled. The primary outcome was cognitive changes after 3 and 12 months post stroke. Among 682 participants, the mean age was 59.35 ± 10.40 years and 72.29% were men. PSCI patients with high systolic blood pressure (SBP ≥140 mm Hg) at 3 m not only had worse cognition as evidenced by MoCA scores at 3 m but also predicted worse scores at 12 m. When participants were stratified into cognitively stable/improved (MoCA score ≥0, 2w vs. 12 m) and cognitively impaired (MoCA score ≤‐2, 2w vs. 12 m), those with high SBP were more likely to be cognitively impaired (OR 2.17, 95%CI 1.12–4.21, *p* < .05) and less likely to be cognitively stable/improved (OR 0.66, 95%CI 0.44–0.99, *p* < .05). SBP more than 140 mm Hg is associated with worse cognitive performance after ischemic stroke. Patients with SBP lower than 140 mm Hg have better cognitive outcome at 3‐month and 1 year after stroke.

## 1 INTRODUCTION

Post stroke cognitive impairment (PSCI) is a common cause of morbidity and disability in cerebrovascular disease.^1^, ^2^ Hypertension is a risk factor for both stroke and dementia.^3^, ^4^, ^5^ Effective treatment of hypertension and cardiac disease help cognitive performance in long the term.^5^, ^6^ However, data from clinical trials designed to demonstrate the benefits of tight blood pressure control on cognition in stroke patients have been inconclusive.^7^, ^8^, ^9^ Intensive systolic blood pressure (SBP) control (less than 120 mm Hg) did not significantly reduce dementia risk but did have a measurable impact on mild cognitive impairment.^10^ Furthermore, permissive hypertension in the immediate post stroke phase is a recognized option in stroke guidelines regarding early penumbra preservation.^11^ Whether this is more or less relevant to cognitively compromised stroke patients with less cerebral plasticity requires further investigation. In this pre‐specified sub‐study of the Impairment of Cognition and Sleep after acute ischemic stroke (ICONS) study, we examined what blood pressure parameters are beneficial to these at risk patients who had cognitive deficits after stroke.

## 2 METHODS

### 2.1 Study design and participants

We used the database of the ICONS study which was conducted at 40 clinical centers in China from August 2015 to April 2018.^12^ In this sub‐study, patients were selected who were within 7 days of confirmed diagnosis of acute ischemic stroke (AIS) on MRI. They completed face to face visits with Montreal Cognitive Assessment (MoCA) tests and blood pressure assessments at 2‐week (2w), 3‐month (3 m), and 12‐month (12 m) after AIS. The blood pressure was measured with the participant in a seated position after 5‐min rest. The position of the automated sphygmomanometer (OMRON Model HEM‐7071, Omron Co. Ltd.) was at the heart level. Measurements were taken two times consecutively on the non‐dominant arm, with 1‐min interval. Blood pressure was recorded as the average of the two measurements. Medications and other medical information were recorded.^12^ The trial was approved by the ethics committee of each medical institution. Written informed consent was obtained from all participants or their legal guardians.

According to 2020 International Society of Hypertension Global Hypertension Practice Guideline,^13^ patients were divided into three groups according to their SBP and DBP ranges at 3 m and 12 m visits: SBP < 130 mm Hg and/or DBP < 85 mm Hg (normal BP), SBP 130–140 mm Hg and/or DBP 85–89 mm Hg (normal‐high BP), SBP ≥140 mm Hg and/or DBP ≥90 mm Hg (high BP).

### 2.2 Outcomes

The safety outcomes in this analysis were similar to those of the ICONS study. The primary efficacy outcome was cognitive decline or improvement at 3 m and 12 m visits compared to the 2w visit, as well as the MoCA scores at 3 m and 12 m visits. MoCA is widely used and has been validated as efficacious in post stroke assessment of cognition due to its sensitivity and specificity in determining mild cognitive impairment (MCI).^14^ Cognitive decline is defined as a reduction in MoCA score of two or more points (one standard deviation, SD),^15^, ^16^ and cognitive improvement as an increase of two or more points.

### 2.3 Statistical analysis

We presented continuous variables as mean ± SD or median with interquartile and categorical variables as percentages. T test or Wilcoxon rank sum test was used for continuous variables and chi‐square test for categorical variables. We compared the factors in these cases with SBP or DBP ranges at 3 m and 12 m visits. All significant covariates (*p* < .05) in the univariable analyses were adjusted in the corresponding multivariable model (Model 3). Demographic characters were analyzed by logistic regression of different models(Model 1: age, sex, and years of education; Model 2: age, sex, years of education and Modified Rankin Scale (MRS); Model 3: age, sex, years of education, MRS, recurrence of stroke and the classification of Trial of Org 10172 in Acute Stroke Treatment (TOAST). Participants with recurrent AIS were removed in this regression analysis as these obviously distorted the figures. Unadjusted and adjusted odds ratios (ORs) and their 95% confidence intervals (CI) were calculated separately. The α level of significance was determined as *p* < .05, two‐sided. All analyses were performed with SAS 9.4 software.

## 3 RESULTS

A total of 682 participants were enrolled in this study. The demographic characters of patients in three groups based on their SBP at 3 m were shown in Table 1. The number of participants in each group were similar. Participants in the high SBP group (≥140 mm Hg) were older, more female, more likely to have recurrent AIS at 3 m. High SBP was associated with worse MoCA scores. Noteworthy, patients with high SBP had worse MoCA scores not only at 3 m but also at 12 m (*p* < .0001), suggesting high SBP at 3 m had a poor prognosis for cognition at 12 m. To validate the relationship between high SBP and cognition at 12 m, we divided participants into three SBP groups at 12 m (Table 2). Participants in the high SBP group (≥140 mm Hg) consistently had worse MoCA scores (*p* < .0001). They were also older and had worse scores of mRS. In terms of DBP, high DBP at 3 m was associated with high BMI and high incidence of bleeding after admission, but not with cognitive performance (Table S1). DBP at 12 m had no correlation with stroke or cognitive function (Table S2).

**TABLE 1:** Demographic, clinical characteristics and outcomes of patients with different SBP ranges at 3 months

	SBP < 130 mm Hg *N* = 232, *n*(%)	130≤SBP < 140 mm Hg *N* = 224, *n*(%)	SBP≥140 mm Hg *N* = 226, *n*(%)	*P* value
Average age (years, mean ± SD) *	58.01±10.48	59.08±10.96	60.99±9.53	.0152
Sex male (%)*	175 (75.43)	169 (75.45)	149 (65.93)	.0330
Years of education (years, mean ± SD)	8.82±2.23	8.96±2.30	8.67±2.30	.4418
Current smokers	95 (40.95)	87 (38.84)	76 (33.63)	.2525
Current drinkers	54 (23.28)	40 (17.86)	49 (21.68)	.3459
Heavy drinkers (>60 g/d)	47 (20.26)	35 (15.63)	43 (19.03)	.4180
BMI (mean±SD)	24.74±3.32	25.19±2.73	25.38±3.34	.1372
History of diabetes	35 (15.09)	42 (18.75)	54 (23.89)	.0559
History of coronary heart disease	20 (8.62)	25 (11.16)	15 (6.64)	.2366
History of atrial fibrillation	7 (3.02)	9 (4.02)	9 (3.98)	.8110
TOAST				.0562
Large artery atherosclerosis	49 (21.12)	59 (26.34)	46 (20.35)	
Cardiogenic embolism	7 (3.02)	12 (5.36)	14 (6.19)	
Small artery occlusion	72 (31.03)	68 (30.36)	72 (31.86)	
Another determined cause	5 (2.16)	0	0	
An undetermined cause	99 (42.67)	85 (37.95)	94 (41.59)	
GAD‐7 at 2w (mean±SD)	2.27±3.32	2.39±3.57	2.31±3.93	.6641
GAD‐7 at 3 m (mean±SD)	1.73±2.93	1.81±3.28	2.06±3.84	.9440
MOCA at 2w (mean±SD) *	21.27±5.92	21.56±5.71	20.00±5.62	.0028
MOCA at 3 m (mean±SD) *	24.37±4.79	24.00±4.83	22.39±5.30	<.0001
MOCA at 12 m (mean±SD) *	24.92±4.66	24.24±5.05	22.77±4.70	<.0001
NIHSS (admission, mean±SD)	3.57±3.45	3.56±2.73	3.62±3.00	.6777
NIHSS (discharge, mean±SD)	1.71±1.92	1.69±1.75	1.96±2.21	.6755
mRS (admission, mean±SD)	0.21±0.55	0.19±0.55	0.29±0.72	.8126
mRS (discharge, mean±SD))	1.03±0.92	0.96±0.84	1.26±1.15	.0504
Recurrence of AIS at 3 m*	6 (2.59)	8 (3.57)	18 (7.96)	.0154
Bleeding after admission	6 (2.59)	2 (0.89)	2 (0.88)	.2174
Recurrence of haemorrhagic stroke at 3 m	0	1 (0.45)	0	.3592

*Abbreviations*: BMI, body mass index; GAD‐7, General Anxiety Disorder‐7; mRS, modified Ranking Scales; NIHSS, National Institutes of Health Stroke Scale;SBP, systolic blood pressure; SD, standard deviation. * *p*<.05.

**TABLE 2:** Demographic, clinical characteristics and outcomes of patients with different SBP ranges at 12 months

	SBP < 130 mm Hg *N* = 244, *n* (%)	130≤SBP < 140 mm Hg *N* = 226, *n* (%)	SBP≥140 mm Hg *N* = 212, *n* (%)	*P* value
Average age (years, mean ± SD) *	57.77±10.16	59.20±10.48	61.32±10.30	.0005
Sex male (%)	65 (26.64)	54 (23.89)	70 (33.02)	.0923
Years of education (years, mean ± SD) *	9.01±2.26	8.92±2.28	8.48±2.25	.0422
Current smokers	97 (39.75)	93 (41.15)	68 (32.08)	.1092
Current drinkers	43 (17.62)	54 (23.89)	46 (21.70)	.2365
Heavy drinkers (>60 g/d)	39 (15.98)	45 (19.91)	41 (19.34)	.4918
BMI (mean±SD)	24.90±3.20	25.23±3.10	25.21±3.15	.6106
History of coronary heart disease	21 (8.61)	25 (11.06)	14 (6.60)	.2558
History of Atrial fibrillation	9 (3.69)	9 (3.98)	7 (3.30)	.9305
TOAST				.6660
Large artery atherosclerosis	56 (22.95)	45 (19.91)	53 (25.00)	
Cardiogenic embolism	12 (4.92)	11 (4.87)	10 (4.72)	
Small artery occlusion	83 (34.02)	66 (29.20)	63 (29.72)	
Another determined cause	3 (1.23)	1 (0.44)	1 (0.47)	
An undetermined cause	90 (36.89)	103 (45.58)	85 (40.09)	
GAD‐7 at 12 m (mean±SD)	1.51±3.04	1.42±2.55	1.81±3.62	.7210
MOCA at 12 m (mean±SD) *	24.95±4.77	24.08±4.86	22.77±4.78	<.0001
mRS at 12 m (mean±SD)*	0.65±0.80	0.72±0.81	0.85±0.85	.0159
Recurrence of AIS at 12 m	17 (6.97)	12 (5.31)	21 (9.91)	.1760
Recurrence of haemorrhagic stroke at 12 m	0	2 (0.88)	1 (0.47)	.3490

*Abbreviations*: BMI, body mass index; GAD‐7, General Anxiety Disorder‐7; mRS, modified Ranking Scales; NIHSS, National Institutes of Health Stroke Scale; SBP, systolic blood pressure; SD, standard deviation. *P<0.05.

To further validate the relationship between different SBP and cognition, we divided participants into the cognitively stable/improved and cognitive impaired groups based on their MoCA scores at 12 m compared to 2w. The cognitively stable/improved group was younger, less likely to have a history of hypertension. The cognitively impaired group was more likely to have previous strokes and an undetermined cause of TOAST type, recurrent AIS and worse mRS scores (Table 3). Recurrent AIS obviously influence the results, after removing them, 632 participants were analyzed in logistic regression of different models, when age, sex, and education were taken into consideration, participants with high SBP were more likely to be cognitively impaired (Table 4, Model 1: OR 2.02, 95%CI 1.06–3.84, *p* = .0322). The strength of the association between high SBP and cognitive impairment was robust when taken in all other factors in addition to age, sex, and education (Table 4, model 3: OR 0.66 (95%CI 0.44–0.99), *p* = .0451; OR 2.17 (95%CI 1.12‐4.21), *p* = .0226, respectively).

**TABLE 3:** Demographic, clinical characteristics and vascular risk factors of patients with different cognitive outcomes

	Cognitively stable/improved *N* = 607, *n* (%)	Cognitively impaired *N* = 75, *n* (%)	*P* value
Average age (years, mean ± SD) *	58.93±10.33	62.77±10.41	.0050
Sex male	444 (73.15)	49 (65.33)	.1538
Years of education (years, mean ± SD)	8.80±2.25	8.96±2.49	.6161
Current smokers	232 (38.22)	26 (34.67)	.5493
Current drinkers	124 (20.43)	19 (25.33)	.3249
Heavy drinkers (>60 g/d)	108 (17.79)	17 (22.67)	.3033
BMI (mean±SD)	25.16±3.18	24.62±2.87	.2432
History of hypertension	342 (56.74)	52 (69.33)	.0316
History of diabetes	116 (19.11)	15 (20.00)	.8536
History of coronary heart disease	53 (8.73)	7 (9.33)	.8622
History of atrial fibrillation	22 (3.62)	3 (4.00)	.8703
TOAST*			.0046
Large artery atherosclerosis	135 (22.24)	19 (25.33)	
Cardiogenic embolism	29 (4.78)	4 (5.33)	
Small artery occlusion	186 (30.64)	26 (34.67)	
Another determined cause	2 (0.33)	3 (4.00)	
An undetermined cause	255 (42.01)	23 (30.67)	
GAD‐7 at 2 weeks (mean±SD)	2.289±3.52	2.587±4.26	.7228
GAD‐7 at 12 months (mean±SD)	1.48±2.84	2.38±4.57	.3482
NIHSS (admission, mean±SD)	3.65±3.14	3.08±2.43	.1804
NIHSS (discharge, mean±SD)	1.78±1,96	1.84±2.01	.8106
mRS (admission, mean±SD)	0.21±0.60	0.27±0.66	.2833
mRS (discharge, mean±SD)	1.09±1.00	1.04±0.88	.9462
mRS at 12 m (mean±SD)*	0.70±0.78	1.03±1.07	.0108
Recurrence of ischemic stroke*	37 (6.10)	13 (17.33)	.0004
Bleeding after admission	10 (1.65)	0	.2628
Recurrence of haemorrhagic stroke at 3 m	1 (0.16)	0	.7250
Recurrence of haemorrhagic stroke at 12 m	2 (0.33)	1 (1.33)	.2152

*Abbreviations*: BMI, body mass index; GAD‐7, General Anxiety Disorder‐7; mRS, modified Ranking Scales; NIHSS, National Institutes of Health Stroke Scale; SD, standard deviation. *p<.05.

**TABLE 4:** Logistic regression analysis of cognitive outcomes at 12 m in first AIS participants

Variables	Events (%)	Model 1 OR	*p*	Model 2 OR	*p*	Model 3 OR	*p*
Cognitively stable/improve							
SBP < 130 mm Hg	146 (63.32)	Reference		Reference		Reference	
130 ≤ SBP < 140 mm Hg	133 (62.15)	0.91 (0.61–1.35)	.6330	0.91 (0.61–1.34)	*p*6306	0.87 (0.58–1.29)	*p*4865
SBP ≥ 140mm Hg^c^	107 (56.02)	0.68 (0.46–1.02)	.0628	0.68 (0.46–1.03)	.0666	0.66 (0.44–0.99)	.0451
Cognitively impaired							
SBP < 130 mm Hg	17 (7.49)	Reference		Reference		Reference	
130 ≤ SBP < 140 mm Hg	15 (7.01)	0.89 (0.43–1.84)	.7496	0.89 (0.43–1.84)	.7517	0.95 (0.45–1.99)	.8824
SBP ≥ 140mm Hg^a b c^	30 (15.71)	2.02 (1.06–3.84)	.0322	2.00 (1.05–3.81)	.0351	2.17 (1.12–4.21)	.0226

a, *p*<.05 in model 1; b, *p*<.05 in model 2; c, *p*<.05 in model 3.

OR1 logistic regression age, sex, and years of education.

OR 2 logistic regression age, sex, years of education, and MRS.

OR 3 logistic regression age, sex, years of education, recurrence of ischemic stroke.

## 4 DISCUSSION

In this study, we have demonstrated the benefits of hypertension control on cognitive performance, when controlled for all other parameters, at 3 m and 12 m post stroke. This is particularly significant when SBP parameters are lowered to less than 140 mm Hg. Interestingly, these benefits were not only realized at 3 m but also extended to 12 m, possibly indicating the benefits of hypertension control early in the course of secondary prevention post stroke.

Hypertension plays a critical role in both salvaging and risking further penumbral damage in AIS depending on when it occurs and whether autoregulation is preserved or compromised. Permissive hypertension in the acute stroke setting is a recognized treatment option with a therapeutic window.^17^ However, lowering blood pressures in the course of an acute stroke and post thrombectomy has demonstrated extension of penumbra and enlargement of stroke bed.^18^, ^19^ Therefore, there is considerable controversy regarding ideal post stroke blood pressure parameters especially in vulnerable patients with cognitive deficits after stroke.^8^, ^9^

There is no correlation between DBP and cognitive performance. SBP is considered a stronger predictor of blood pressure related outcomes than DBP.^20^, ^21^ A meta‐analysis has demonstrated that SBP has a linear effect on cognitive decline, and lower SBP levels are associated with slower cognitive decline of later life.^22^

A smaller study recruited patients with first‐ever stroke who achieved SBP in the categories ≤125 mm Hg, ≤140 mm Hg, and ≤160 mm Hg, SBP reduction of ≥10 mm Hg, and DBP reduction of ≥5 mm Hg. It showed a potential beneficial effect of blood pressure control on cognitive function (*p* = .070–1.0), suggesting longer follow‐up and/or larger sample size were needed to demonstrate significance.^23^ A sub‐analysis of the Secondary Prevention of Small Subcortical Strokes Study (SPS3) trial assessed the effects of blood pressure control and single/dual antiplatelet treatment on cognition. It recruited patients with recent (within 6 months) symptomatic lacunar infarcts and tested their blood pressure and cognition annually. It didn't show significant changes in cognitive function over time between assigned antiplatelet groups (*p* = .858) or between assigned blood pressure target groups (SBP 130–149 mm Hg vs. < 130 mm Hg, *p* = .520),^24^ suggesting targeted stratification of SBP groups and earlier intervention are critical .

Stroke scales are designed to rapidly localize the presence or risk of stroke to large and small vessel territories. Cognitive tests are designed to measure function at targeted domains of the brain. Cognitive measures such as MoCA are likely to capture more subtle losses of function than stroke scales and are designed to show progressive performance change rather than acute risk. While stroke studies have focused on the presence of recurrent stroke, a recent study indicated that cognitive measures correlated more with blood pressure and cardiac parameters than recurrent TIA or stroke events.^25^ Hypertension at 3 m is associated with higher recurrence rate of AIS within 3 m and worse long term mRS scores at 12 m. Recurrent AIS can impact negatively on cognition. However, after excluding the effects of recurrent AIS, SBP still has significant impact on cognitive performance (Table 4, model 3).

There are several limitations of the study. First, this is a prospective observational study. Age, sex, and education levels were not tightly controlled for study groups. Since all of them can affect cognition, we used logistic regression analysis (model 1) to discount their effects. Second, blood pressure is likely to fluctuate in the period of 1 year. It has been shown that the fluctuations in blood pressure have predictive values in patients with stroke, a potential risk factor for cognitive decline in dementia. We tried to limit this fluctuation by multiple analysis at 3 m and 12 m. The results from these two time points are consistent. Third, stroke‐like symptoms could mimic stroke and compromise the accuracy of diagnosis of PSCI. We required MRI imaging evidence of stroke to exclude stroke mimics for this study. Future studies should attempt to correlate MRI stroke region with cognitive performance and potentially localize at risk subtypes.

In conclusions, this study has shown that hypertension (SBP≥140 mm Hg), as an independent risk factor, has detrimental effects on cognitive function after initial AIS. Navigating appropriate blood pressure parameters in PSCI patients with various risk factors for stroke and dementia is difficult. However, determining appropriate parameters for this at risk population may provide effective protection against further decline.

## AUTHOR CONTRIBUTIONS

X.L., J.S. and Y.W. designed the study. Y.W., S.L. and J.S. did the scientific literature search. Y.W., S.L., X.L., Y.W. collected data. Y.W., S.L., Y.P., M.W., J.S. and Y.W. analyzed data. Y.W., S.L., Y.P., J.S. and Y.W. interpreted data. Y.W. and M.W. created the tables. Y.W., S.L. and J.S. wrote and all authors edited the report. All authors read and approve the manuscript.

## CONFLICT OF INTEREST

Authors report no conflict of interest.

## Supporting information

Supporting information.
