# Supplementary material for: The effects of blood pressure on post stroke cognitive impairment: BP and PSCI
Source: J Clin Hypertens (Greenwich). 2021 Nov 20;23(12):2100–5. doi: 10.1111/jch.14373 (PMC8696235; doi:10.1111/jch.14373)
Supplement: Supplementary file 1 — Supporting information. [file JCH-23-2100-s001.docx]

SUPPLEMENTAL MATERIAL

Supplemental Table 1：Demographic, clinical characteristics and outcomes of patients with different DBP ranges at 3 months

| Variables | DBP < 85mmHg  N=441, n(%) | 85≤DBP < 90mmHg  N=119, n(%) | DBP≥90mmHg  N=122, n(%) | P value |
| --- | --- | --- | --- | --- |
| Average age (years, mean+SD) | 59.47±10.74 | 59.51±10.09 | 58.77±9.43 | 0.7534 |
| Gender male (%) | 323(73.24) | 88(73.95) | 82(67.21) | 0.3804 |
| Years of education (years, mean+SD) | 8.83±2.24 | 8.70±2.43 | 8.87±2.24 | 0.8144 |
| Current smokers | 170(38.55) | 37(31.09) | 51(41.80) | 0.2006 |
| Current drinkers | 90(20.41) | 23(19.33) | 30(24.59) | 0.5373 |
| Heavy drinkers(＞60g/d) | 75(17.01) | 21(17.65) | 29(23.77) | 0.2270 |
| BMI (mean+SD) * | 24.87±3.03 | 25.17±3.12 | 25.91±3.48 | 0.0211 |
| History of Diabetes | 84(19.05) | 27(22.69) | 20(16.39) | 0.4586 |
| History of coronary heart disease | 41(9.30) | 8(6.72) | 11(9.02) | 0.6761 |
| History of Atrial fibrillation | 12(2.72) | 8(6.72) | 5(4.10) | 0.1149 |
| TOAST |  |  |  | 0.4385 |
| large artery atherosclerosis | 99(22.45) | 21(17.65) | 34(27.87) |  |
| cardiogenic embolism | 18(4.08) | 8(6.72) | 7(5.74) |  |
| small artery occlusion | 139(31.52) | 39(32.77) | 34(27.87) |  |
| another determined cause | 5(1.13) | 0 | 0 |  |
| an undetermined cause | 180(40.82) | 51(42.86) | 47(38.52) |  |
| GAD-7 at 2w (mean+SD) | 2.34±3.44 | 2.08±3.14 | 2.50±4.53 | 0.5654 |
| GAD-7 at 3m (mean+SD) | 1.83±3.24 | 1.93±3.03 | 1.93±4.07 | 0.1167 |
| MOCA at 2w (mean+SD) | 21.04±5.88 | 20.64±5.70 | 20.88±5.53 | 0.6450 |
| MOCA at 3m (mean+SD) | 23.82±4.97 | 22.97±5.36 | 23.39±4.94 | 0.2577 |
| NIHSS (admission, mean+SD) | 3.66 ±3.27 | 3.64±2.72 | 3.24±2.62 | 0.3324 |
| NIHSS (discharge, mean+SD) * | 1.81±1.92 | 1.96±2.00 | 1.53±2.11 | 0.0246 |
| MRS (admission, mean+SD) | 0.17±0.49 | 0.34±0.86 | 0.24±0.69 | 0.2680 |
| MRS (discharge, mean+SD) | 1.05±0.94 | 1.24±1.08 | 1.03±1.04 | 0.2122 |
| Recurrence of ischemic stroke at 3m | 17(3.85) | 9(7.56) | 6(4.92) | 0.2348 |
| Bleeding after admission* | 4(0.84) | 1(0.97) | 5(4.85) | 0.0080 |
| Recurrence of haemorrhagic stroke at 3m | 1(0.21) | 0 | 0 | 0.8052 |

Abbreviations: DBP, diastolic blood pressure; BMI, body mass index; TIA, transient ischemic attack; GAD-7, General Anxiety Disorder-7; NIHSS, National Institutes of Health Stroke Scale; mRS, modified Ranking Scales; SD, standard deviation. * P＜0.05

Supplemental Table 2：Demographic, clinical characteristics and outcomes of patients with different DBP ranges at 12 months

| Variables | DBP < 85mmHg  N=476, n(%) | 85≤DBP < 90mmHg  N=103, n(%) | DBP≥90mmHg  N=103, n(%) | P value |
| --- | --- | --- | --- | --- |
| Average age (years, mean+SD) | 59.29±10.56 | 60.04±8.93 | 58.93±11.03 | 0.6862 |
| Gender male (%) | 346(72.69) | 68(66.02) | 79(76.70) | 0.2166 |
| Years of education (years, mean+SD) | 8.85±2.28 | 8.85±2.35 | 8.59±2.17 | 05973 |
| Current smokers | 175(36.76) | 42(40.78) | 41(39.81) | 0.6767 |
| Current drinkers | 96(20.17) | 18(17.48) | 29(28.16) | 0.1254 |
| Heavy drinkers(＞60g/d) | 82(17.23) | 16(15.53) | 27(26.21) | 0.0742 |
| BMI (mean+SD) | 25.12±3.26 | 24.92±2.67 | 25.23±3.07 | 0.8624 |
| History of Diabetes | 92(19.33) | 18(17.48) | 21(20.39) | 0.8624 |
| History of coronary heart disease | 39(8.19) | 12(11.65) | 9(8.74) | 0.5321 |
| History of Atrial fibrillation | 15(3.15) | 3(2.91) | 7(6.80) | 0.1845 |
| TOAST |  |  |  |  |
| large artery atherosclerosis | 109(22.90) | 23(22.33) | 22(21.36) |  |
| cardiogenic embolism | 22(4.62) | 5(4.85) | 6(5.83) |  |
| small artery occlusion | 154(32.35) | 26(25.24) | 32(31.07) |  |
| another determined cause | 5(1.05) | 0 | 0 |  |
| an undetermined cause | 186(39.08) | 49(47.57) | 43(41.75) |  |
| GAD-7 at 12m (mean+SD) | 1.62±3.08 | 1.64±3.41 | 1.31±2.76 | 0.4146 |
| MoCA at 12m (mean+SD) | 24.19±4.87 | 23.33±4.77 | 23.69±5.0 | 0.1130 |
| mRS at 12m (mean+SD) | 0.72±0.83 | 0.88±0.92 | 0.66±0.68 | 0.1882 |
| Recurrence of AIS at 12m | 34(7.14) | 10(9.71) | 6(5.83) | 0.5418 |
| Recurrence of haemorrhagic stroke at 12m | 2(0.42) | 1(0.97) | 0 | 0.5705 |

Abbreviations: DBP, diastolic blood pressure; BMI, body mass index; TIA, transient ischemic attack; GAD-7, General Anxiety Disorder-7; NIHSS, National Institutes of Health Stroke Scale; mRS , modified Ranking Scales; SD, standard deviation. * P＜0.05
